# Low‐Dose Tamoxifen Induces Significant Bone Formation in Mice

**DOI:** 10.1002/jbm4.10450

**Published:** 2021-01-20

**Authors:** Zhihui Xie, Cody McGrath, Jeyantt Sankaran, Maya Styner, Sarah Little‐Letsinger, Amel Dudakovic, Andre J van Wijnen, Janet Rubin, Buer Sen

**Affiliations:** ^1^ Department of Medicine University of North Carolina Chapel Hill NC USA; ^2^ Department of Orthopedic Surgery and Biochemistry and Molecular Biology Mayo Clinic Rochester MN USA

**Keywords:** CONDITIONAL KNOCKOUT, OSTEOCLAST, μCT

## Abstract

Use of the selective estrogen receptor modulator Tamoxifen (TAM) is a mainstay to induce conditional expression of Cre recombinase in transgenic laboratory mice. To excise β‐catenin^*fl/fl*^ in 28‐day‐old male and female Prrx1‐CreER/β‐catenin^*fl/fl*^ mice (C57BL/6), we utilized TAM at 150 mg/kg; despite β‐catenin knockout in MSC, we found a significant increase in trabecular and cortical bone volume in all genders. Because TAM was similarly anabolic in KO and control mice, we investigated a dose effect on bone formation by treating wild‐type mice (WT C57BL/6, 4 weeks) with TAM (total dose 0, 20, 40, 200 mg/kg via four injections). TAM increased bone in a dose‐dependent manner analyzed by micro–computed tomography (μCT), which showed that, compared to control, 20 mg/kg TAM increased femoral bone volume fraction (bone volume/total volume [BV/TV]) (21.6% ± 1.5% to 33% ± 2.5%; 153%, *p* < 0.005). With TAM 40 mg/kg and 200 mg/kg, BV/TV increased to 48.1% ± 4.4% (223%, *p* < 0.0005) and 58% ± 3.8% (269%, *p* < 0.0001) respectively, compared to control. Osteoblast markers increased with 200 mg/kg TAM: *Dlx5* (224%, *p* < 0.0001), *Alp* (166%, *p* < 0.0001), *Bglap* (223%, *p* < 0.0001), and *Sp7* (228%, *p* < 0.0001). Osteoclasts per bone surface (Oc#/BS) nearly doubled at the lowest TAM dose (20 mg/kg), but decreased to <20% control with 200 mg/kg TAM. Our data establish that use of TAM at even very low doses to excise a floxed target in postnatal mice has profound effects on trabecular and cortical bone formation. As such, TAM treatment is a major confounder in the interpretation of bone phenotypes in conditional gene knockout mouse models. © 2020 The Authors. *JBMR Plus* published by Wiley Periodicals LLC. on behalf of American Society for Bone and Mineral Research.

## Introduction

Tamoxifen (TAM)‐inducible Cre Estrogen Receptor is one of the most widely used inducible systems for postnatal gene manipulation.^(^ ^1^, ^2^ ^)^ This system fuses DNA encoding a modified Cre protein with that for an estrogen receptor containing a mutated ligand binding domain.^(^ ^3^, ^4^ ^)^ The translated CreER recombinase binds to the cytoplasmic heat shock protein 90 (HSP90). Tamoxifen disrupts the interaction between HSP90 and CreER to cause nuclear translocation of CreER. Once intranuclear, Cre interacts with flanking loxP sequences (ie, 34–base pair repeats) near target genes (floxed alleles). Cre‐mediated recombination then excises either critical parts of the target gene to excise the intervening DNA and inactivate gene function.

Previously, we found that β‐catenin prevented both osteogenic and adipogenic lineage commitment.^(^ ^5^ ^)^ To further investigate the role of β‐catenin on bone remodeling in vivo, we employed the CreER system for conditional and inducible removal of β‐catenin to bypass the phenotypes observed by constitutive gene KO at early stages in embryo development.

In our experimental design, we were cognizant that TAM is an estrogen modulator which influences bone metabolism and contributes to increased bone formation.^(^ ^6^, ^7^, ^8^ ^)^ A recent study indicated that a high dose of TAM (100 mg/kg × 4 injections, 400 mg/kg total) considerably increased trabecular bone formation in male mice, but that a lower dose (10 mg/kg × 4 injections, 40 mg/kg total) did not significantly change any formation‐related parameters.^(^ ^9^ ^)^ Further, animal age, sex, and TAM dosing frequency might also be pertinent to TAM delivery designed with an effort to avoid effects on bone formation. Gene inactivation studies use TAM doses between 70 mg/kg and 100 mg/kg × 3 or 5 (total 225 mg/kg to 500 mg/kg). Hence, to permit comparisons with other genetic loss‐of‐function models,^(^ ^10^, ^11^, ^12^, ^13^, ^14^, ^15^, ^16^, ^17^, ^18^, ^19^ ^)^ we initially studied lower doses: 30 mg/kg given either 5 times or 2 times every other day (total 150 mg/kg and 60 mg/kg, respectively) to induce conditional knockout of β‐catenin in MSC using a Prrx1 promoter controlled inducible Cre/ERT2 driver.

Our main finding is that bone formation significantly increased in both control mice (Prrx1‐Cre/ERT2) or mice with the conditional β‐catenin^*fl/fl*^ allele, and that reducing the total dose from 150 to 60 mg/kg in Prrx1‐Cre/ERT2 mice did not change the outcome. Furthermore, wild‐type (WT) C57BL/6 mice treated with different TAM doses (0, 5, 10, 50 mg/kg × 4) exhibited a dose‐dependent increase in bone formation, with inhibition of osteoclast formation at the higher doses. Importantly, even the lowest dose, 20 mg/kg total, significantly increased bone quantity. Because of the relatively high doses of TAM (>30 mg/kg) required for effective Cre‐mediated recombination, and the increased bone accrual observed with low doses of TAM, at a pragmatic level it may be difficult, if not impossible, to use a TAM dose for inducible conditional knockout without confounding bone phenotyping.

## Subjects and Methods

### Animals

Animal experiments were approved by UNC and Mayo IACUCs. B6.129S4‐*Gt(ROSA)26Sor* ^*tm1Sor*^/J (#003474), B6.Cg‐Tg (Prrx1‐cre/ERT2, EGFP)/1Smkm/J (#029211), and B6.129‐Ctnnb^*1tm2km*^/KnwJ (βCat^*fl/fl*^, #004152) and WT C57BL/6 (#000664) were obtained from The Jackson Laboratory (Bar Harbor, ME, USA). The Prrx1CreER strain was crossed with βCat^*fl/fl*^ mice to generate the Prrx1Cre+/βCat^*fl/fl*^ model. Mice containing a conditional Ezh2^*fl/fl*^ allele flanking the SET domain were obtained from a Mutant Mouse Regional Resource Center (B6;129P2‐Ezh2tm1Tara/Mmnc University of North Carolina, Chapel Hill, NC, USA). Ezh2^*fl/fl*^ mice were crossed with αSMA‐Cre/ERT2 (a kind gift from Ivo Kalajzic, UConn Health, CT).^(^ ^20^ ^)^

### Experimental design

Mice were randomly allocated into two groups: TAM‐injected group and vehicle (VEH)‐injected group (Ctrl) for Prrx1‐Cre/βCat^*fl/fl*^, Prrx1‐Cre/ERT2, and C57BL/6. Tamoxifen (T5648; Sigma‐Aldrich, St. Louis, MO, USA; 50 mg/mL of stock solution [50 mg of 4‐OHT powder in 1 mL of ethanol] diluted in corn oil at varying doses, 100 μL/each dose) or corn oil (100 μL) were warmed to 37°C and injected into the peritoneal cavity beginning at 4 weeks. After injection, the site was massaged to facilitate diffusion, and the injection site was alternated between left and right sides. Mice were euthanized 4 weeks after start of an experiment for femur and tibia harvest.

For Ezh2‐related studies, Sma‐Cre/ERT2‐positive control (Ezh2^*wt/wt*^: Sma‐Cre/ERT2+), Ezh2 floxed control (*Ezh2* ^*fl/fl*^: *Sma‐Cre/ERT2–*), and Ezh2 conditional knockouts (*Ezh2* ^*fl/fl*^: *Sma‐Cre/ERT2+*) male mice were injected with TAM (75 μg/g mouse weight) or DMSO diluted in corn oil (100 μL/mouse) three times between weeks 3 and 4 of age via intraperitoneal injections. Mice were euthanized 3 weeks after TAM dosing began, and femurs assessed by μCT analysis.

### Histology

Femurs were fixed in 4% paraformaldehyde at 4°C under constant agitation × 1 day before decalcification (14% EDTA solution, changed daily) at 4°C under constant agitation for 5 days. After PBS washing for 2 hours, specimens were transferred to 70% ethanol. Femurs were sectioned at 5 μm and stained with H&E. For osteoclast quantification, sections were stained for tartrate‐resistant acid phosphatase (TRAP) with Fast Green (Sigma‐Aldrich; F7252‐5G) background stain. Imaging was performed via the Olympus X81 at ×10 magnification (Olympus, Waltham, MA, USA). Analysis for osteoclast number/bone surface was performed using the open source applications Image J software (NIH, Bethesda, MD, USA; https://imagej.nih.gov/ij/) after staining for TRAP.^(^ ^21^ ^)^

### Bone microarchitecture via micro–computed tomography

Fixed femurs were imaged via micro–computed tomography (μCT). Bone morphology μCT parameters were quantified (Scanco Medical, Wayne, PA, USA) as described (resolution = 12 μm, E = 55 kVa, I = 145 μA).^(^ ^22^ ^)^ Trabecular parameters included trabecular bone volume (Tb.BV), trabecular bone volume fraction (Tb.BV/TV), trabecular number (Tb.N, 1/mm), trabecular thickness (Tb.Th, mm), and trabecular spacing (Tb.Sp, mm). Cortical parameters included cortical area (Ct.Ar, mm^2^), cortical thickness (Ct.Th, mm), and cortical bone volume fraction (Ct.Ar/Tt.Ar). A threshold for each slice was set exclusively for cortical and trabecular bone using an automated script. The reconstructed 3D images were used to quantify microarchitecture.

### Real‐time PCR

Quantitative PCR was performed as described.^(^ ^23^ ^)^ Briefly, 1 μg of mRNA from whole tibia was reverse‐transcribed (RT). Standards and samples were run in triplicate. PCR products were normalized to 18 S amplicons (RT) and standardized on a dilution curve from RT sample. Primers: Alpl, Sp7, Bglap, and 18S primers were as in Bouxsein and colleagues.^(^ ^23^ ^)^

### Statistical analysis

Results are expressed as mean ± SD unless noted otherwise. Statistical significance was evaluated by one‐way analysis of variance or *t* test as appropriate (GraphPad Prism; GraphPad Software, Inc., La Jolla, CA, USA). All experiments were replicated at least three times to assure reproducibility.

## Results

**Fig 1:**
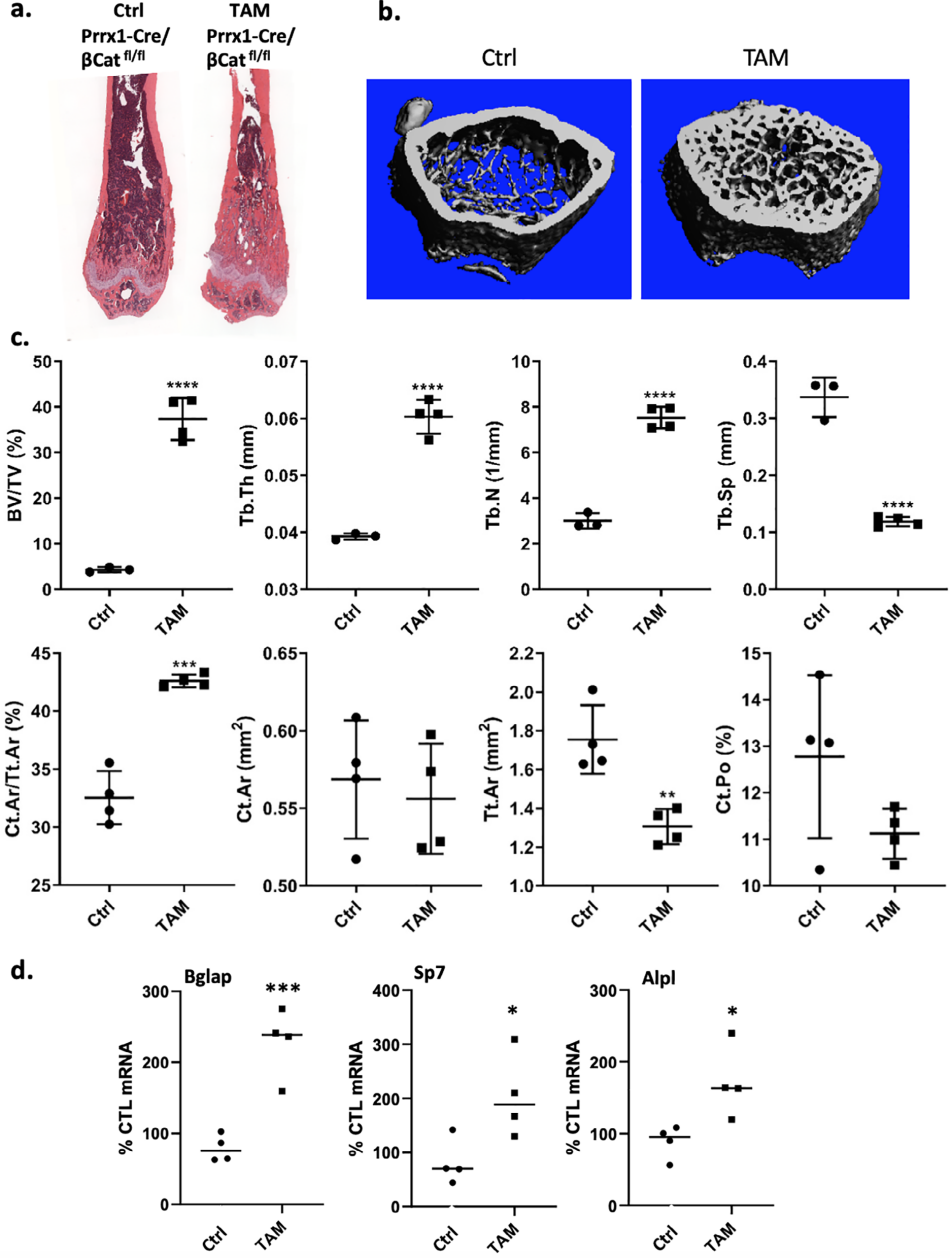
Bone parameters significantly increased after TAM treatment in Prrx1‐Cre/βCat^*fl/fl*^ mice. Female mice (*n* = 5 group) were injected with vehicle or TAM (100 μL/mouse, 30 mg/kg × 5; total dose 150 mg/kg dosed over 10 days) beginning at 4 weeks of age and bones harvested at 8 weeks. (*A*) Paraffin‐embedded H&E‐stained tibia sections. (*B*) μCT reconstructions of femurs demonstrate more bone in TAM versus VEH; each point represents a separate mouse. (*C*) Femoral μCT parameters for trabecular and cortical sites. (*D*) Real‐time PCR for bone formation marker genes with RNA from tibial bone (±SE). Animal data is presented as means ± SD; statistical significance indicated on plots: *p < .05, **p < 0.01, ***p < 0.001 and ****p < 0.0001.

We initiated studies in which we excised a floxed β‐catenin allele in cells positive for Prrx1 using a conditional Prrx1‐Cre/ERT2 driver known to be effective in mice less than 4 weeks old.^(^ ^24^ ^)^ Prrx1 is expressed throughout the embryonic limb bud mesenchyme and contributes to formation of the growth plate and the bone collar, as well as secondary ossification centers.^(^ ^25^ ^)^ We validated the activity of Cre recombinase by crossing Prrx1‐cre/ERT2 mice to Cre reporter strains, Rosa26^LacZ^. Supplemental Fig. 1 shows βGal stained cells in the femur of Prrx1‐Cre/Rosa26^LacZ^ of mice dosed with TAM. Upon crossing of the βCat^*fl/fl*^ (The Jackson Laboratory) allele with the conditional Prrx1‐Cre/ERT2 driver to obtain Prrx1‐Cre/βCat^*fl/fl*^ mice, we induced the excision by TAM treatment. Many studies effectively activate CreER‐driven conditional gene knockout using a high dose of TAM (a total of 225 to 500 mg/kg from 70 to 100 mg/kg/day for 3 to 5 days) to validate the genetic loss‐of‐function results.^(^ ^10^, ^11^, ^12^, ^13^, ^14^, ^15^, ^16^, ^17^, ^18^, ^19^ ^)^ Previous studies have indicated that TAM doses of 10 mg/kg × 4 given over 4 days (total 40 mg/kg) avoid bone phenotypic effects that are apparent at higher doses.^(^ ^9^ ^)^ To balance the known effects of TAM on bone formation with the efficiency of Cre excision of floxed alleles, we treated two groups of five female Prrx1‐Cre/βCat^*fl/fl*^ mice with either corn oil or TAM (100 μL/mouse, 30 mg/kg × 5; total dose 150 mg/kg dosed every other day) beginning at 4 weeks of age. Analysis by μCT verified that TAM dramatically induced BV/TV (158.5%), Tb.Th (42.2%), Tb.N (86%), and reduced Tb.Sp (95.6%) (Fig. 1*B*,*C* where each point represents a separate mouse). Cortical bone at the mid‐diaphysis (Ct.Ar/Tt.Ar) was significantly increased. The results of real‐time PCR were consistent with those of histologic microscopy and μCT, demonstrating that gene expression of bone markers (*Bglap*, *Sp7*, *Alpl*, and *Dlx5*) were significantly increased in the bones from TAM‐induced mice compared to Ctrl mice (Fig. 1*D*). Femurs and tibias prepared for histology showed that TAM caused substantial increases in femoral trabecular and cortical bone volume in all TAM‐treated mice shown as whole bone mounts (Fig. 1*A*, Supplemental Fig. 2).

**Fig 2:**
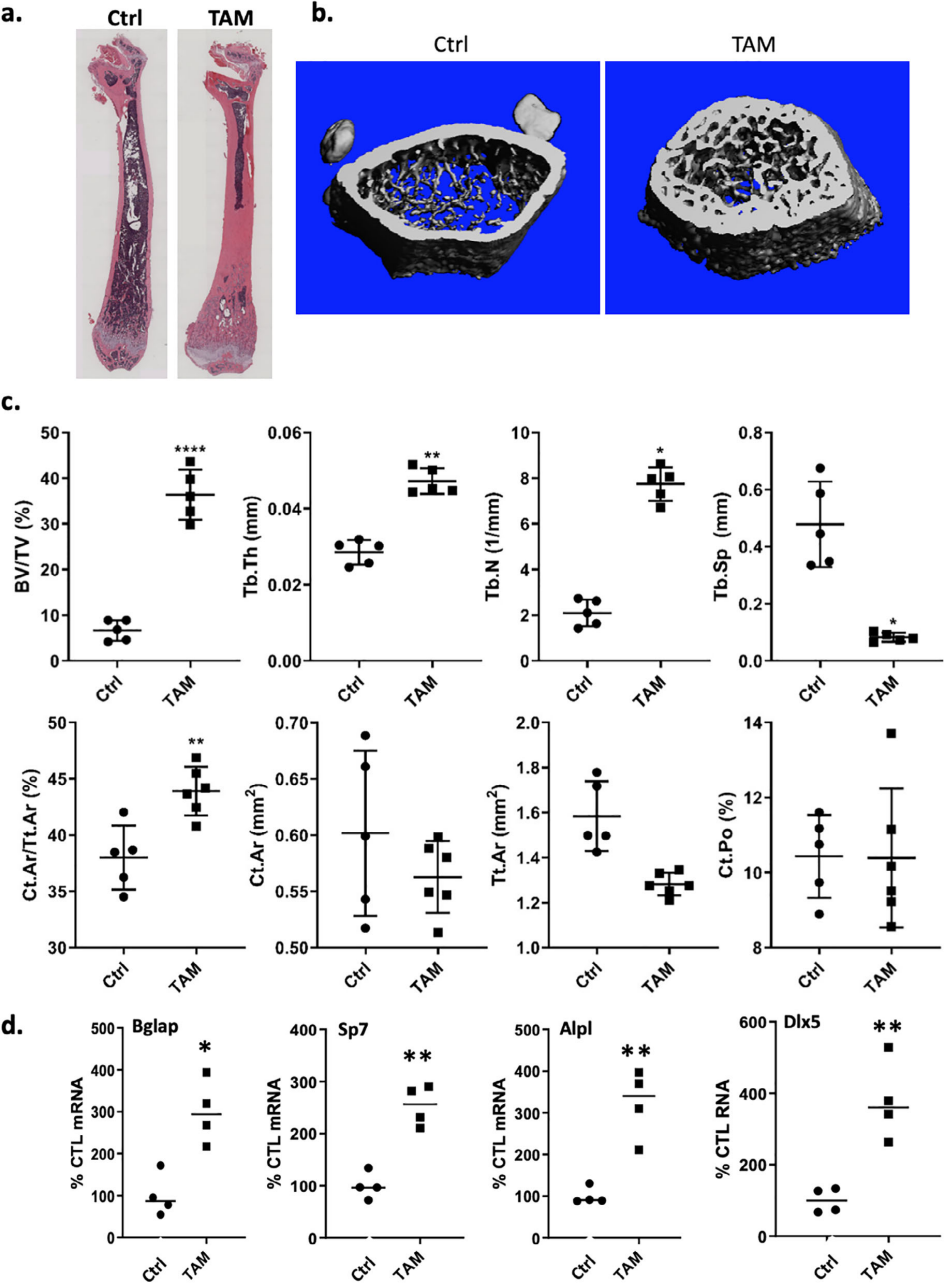
Bone parameters in TAM treated Prrx1‐Cre/ERT2 male mice recapitulate anabolic effect in females. Male Prrx1‐cre/ERT2 mice (*n* = 3) were treated with vehicle or TAM (30 mg/kg i.p. × 5; total dose 150 mg/kg) starting at 4 weeks of age with harvest at 8 weeks. (*A*) H&E‐stained tibia. (*B*) μCT reconstructions of the femurs. (*C*) μCT parameters for trabecular and cortical bones. (*D*) Real‐time PCR for bone formation marker genes with total RNA from tibial bone. Statistical significance is indicated as follows: *p < .05, **p < 0.01, and ****p < 0.0001.

Although this pilot experiment appeared to suggest a potential effect of β‐catenin deletion in Prrx1‐expressing cells on bone formation, we were surprised by the magnitude of the effect and performed additional experiments to account for the biological effects of TAM. To eliminate estrogen‐related issues in female mice, the follow‐up study used male Prrx1‐Cre/ERT2 mice (from the same litters as in the first experiment) and analyzed two treatment groups of five mice each that were dosed with 30 mg/kg TAM × 5 or corn oil × 5. The mice treated with TAM showed significant increases in bone formation, comparable to that in TAM‐induced female β‐catenin knockout littermates. Histological staining showed that TAM induced a similar increase of bone formation (Figs. 1*A* and 2*A*, Supplemental Fig. 3). μCT parameters revealed significant increases for trabecular BV/TV (+137.9%), trabecular thickness (Tb.Th +49.3%), trabecular number (Tb.N + 114.5%), and a significant decrease in Tb.Sp (+140.9%) (Fig. 2*B*,*C*). Further, confirming histologic images, cortical bone volume fracture at the mid‐diaphysis (Ct.Ar/Tt.Ar) was significantly increased. In male mice treated with TAM, osteogenic genes (*Bglap*, *Sp7*, *Alpl*, *Dlx5*) were also significantly upregulated as measured by real‐time PCR on bone samples, with more threefold increases in osteogenic gene expression (Fig. 2*D*).

**Fig 3:**
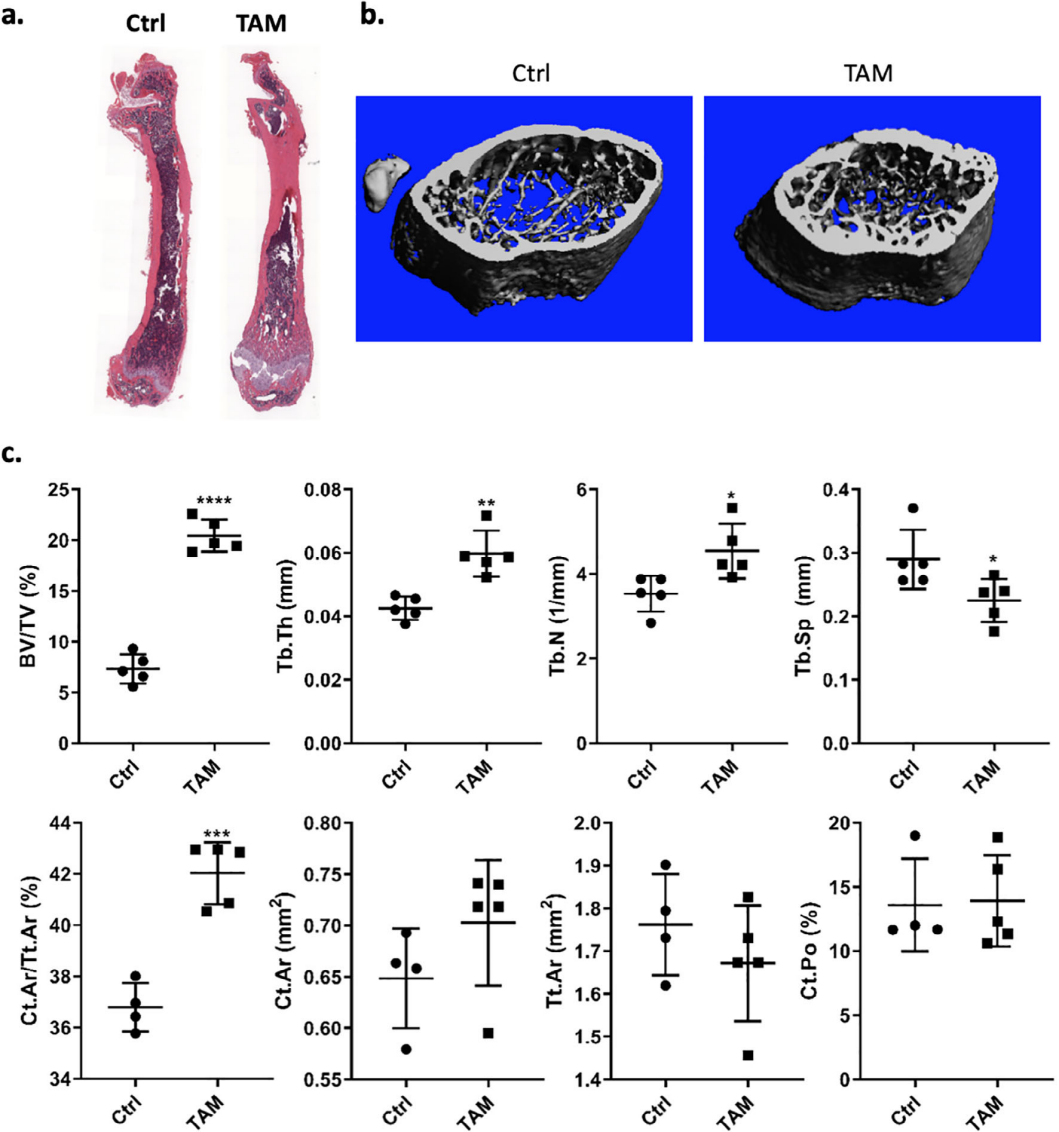
Bone parameters significantly increased after two doses of TAM treatment in Prrx1‐Cre/ERT2 male mice. Male mice (*n* = 4/group) treated with vehicle or TAM (30 mg/kg × 2; total dose 60 mg/kg) at 4 weeks of age with bones harvest at 8 weeks. (*A*) H&E‐stained tibia. (*B*) μCT reconstructions of the femurs. (*C*) μCT parameters for trabecular and cortical bones. Statistical significance is indicated as follows: *p < .05, **p < 0.01, ***p < 0.001 and ****p < 0.0001.

We addressed whether a reduction in the total amount and number of doses of TAM would increase bone quantity. In these experiments, Prrx1‐Cre/ERT2 male mice were treated with two injections of 30 mg/kg (total of 60 mg/kg) within 1 week. μCT parameters showed smaller effects than with 150 mg/kg, but increases were still significant: BV/TV (+94.5%), Tb.Th (+33.6%), Th.N (+24.9%), and decreased Tb.Sp (−25.1%) (Fig. 3*B*). This dosing scheme increased bone formation viewed on whole‐mount femur histology (Fig. 3*A*, Supplemental Fig. 4). Cortical bone at the mid‐diaphysis (Ct.Ar/Tt.Ar) was, as well, significantly increased.

**Fig 4:**
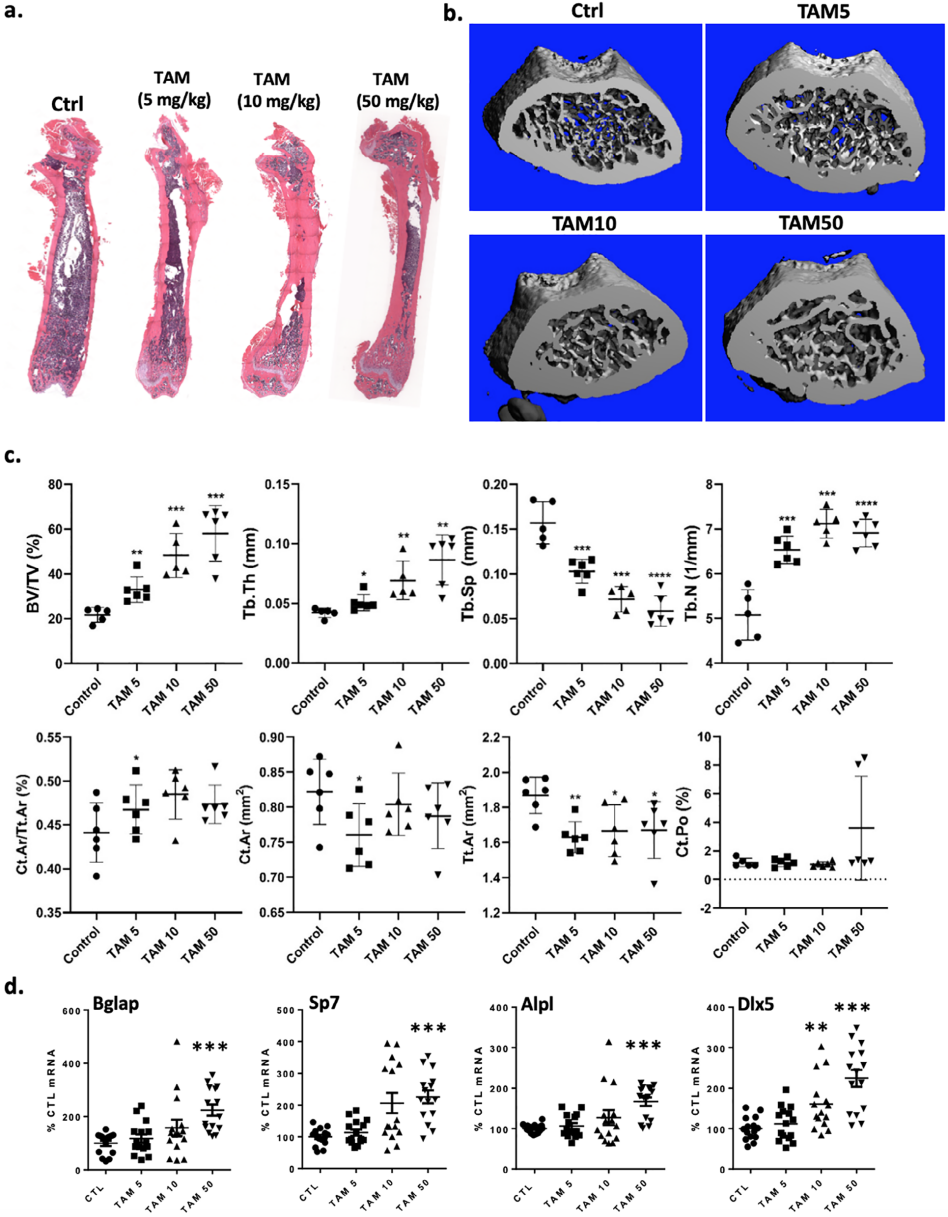
Dose‐dependent increase in trabecular and cortical bone due to TAM in C57BL/6 mice. Male (*n* = 5/group) were treated with vehicle or TAM (5 or 10 or 50 mg/kg × 4; total dose 20 or 40 or 200 mg/kg dosed over 8 days) at 4 weeks of age with bones collected at 8 weeks. (*A*) H&E‐stained tibia. (*B*) μCT reconstructions of the femurs. (*C*) μCT parameters for trabecular and cortical bones. (*D*) Real‐time PCR for bone formation marker genes with total tibial bone RNA; there are at least three technical replicates from each of five mice. Statistical significance is indicated as follows: **p < 0.01 and ***p < 0.001.

With varied dosing regimens of TAM showing effects in both male and female mice, we then performed dose response experiments on WT mice. Four week‐old male C57BL/6 mice were treated with vehicle (Ctrl, corn oil × 4 doses) or TAM (× 4 doses) at total doses of 20, 40 and 200 mg/kg. Mice were euthanized at 8 weeks of age (3 weeks after the last dose). Histology (Fig. 4*A*, Supplemental Fig. 5) and μCT revealed that the lowest total dose of TAM (20 mg/kg) caused significant increases in trabecular bone, increasing dose‐dependently thereafter, and cortical bone Ct.Ar/Tt.Ar (Fig. 4*A*–*C*, *p* < 0.05). Expression of the osteoblast‐related gene *Dlx5* increased 160% (*p* < 0.005) at 10 mg/kg dosing rising to 224% (*p* < 0.0001) at 50 mg/kg, compared to control. Other osteoblast genes also showed a trend toward increased expression, but only achieved significance at the highest TAM dose: +166% for *Alp* (*p* < 0.0001), +223% for *Bglap* (*p* < 0.0001), and +228% for *Sp7* (*p* < 0.0001) compared to control (Fig. 4*D*).

**Fig 5:**
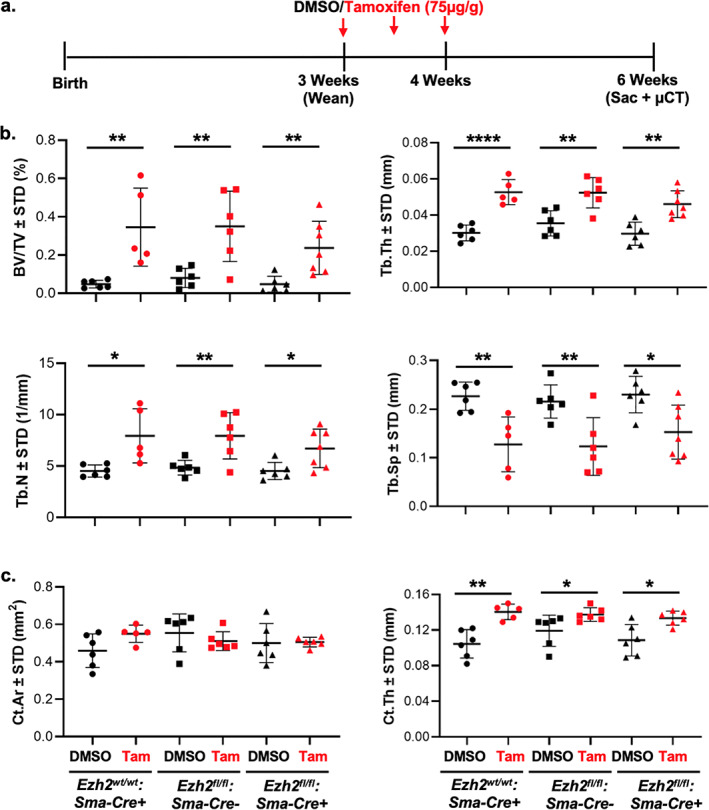
Independent validation of TAM effect on bone in three additional genotypes of CrER transgenic mice. Three genotypes (Ezh2^*wt/wt*^: Sma‐Cre/ERT2+, Ezh2^*fl/fl*^: Sma‐Cre/ERT2–, and Ezh2^*fl/fl*^: Sma‐Cre/ERT2+) of male mice were injected TAM (75 μg/g mouse weight) or DMSO diluted in corn oil (100 μL/mouse) three times between weeks 3 and 4 of age by intraperitoneal injections. Mice were euthanized at 6 weeks of age and femurs assessed by μCT analysis. Experimental design (*A*), trabecular (*B*), and cortical (*C*) bone parameters as measured by μCT assessment (*n* = 5 to 7, mean ± SD). Statistical significance is indicated as follows: *p < .05, **p < 0.01, and ****p < 0.0001.

The potent effects of TAM on bone microarchitecture at the University of North Carolina was confirmed with those obtained independently in a second laboratory at Mayo Clinic. In these parallel studies, three strains of male mice (Ezh2^*wt/wt*^;Sma^_^Cre/ERT2^+^, Ezh2^*fl/fl*^;Sma^_^Cre/ERT2^−^, and Ezh2^*fl/fl*^;Sma‐Cre/ERT2^+^) were treated with TAM at 75 mg/kg × 3 over 1 week (total dose 225 mg/kg, Fig. 5*A*). Shown in Fig. 5*B*, changes in BV/TV, Tb.Th, Tb.N, and Tb.Sp established that TAM was associated with increase in trabecular bone formation in all strains, regardless of presence or absence of floxed genes or presence of Cre. In addition, the cortical thickness increased in all animals measured 3 weeks after the first TAM dose (Fig. 5*C*).

**Fig 6:**
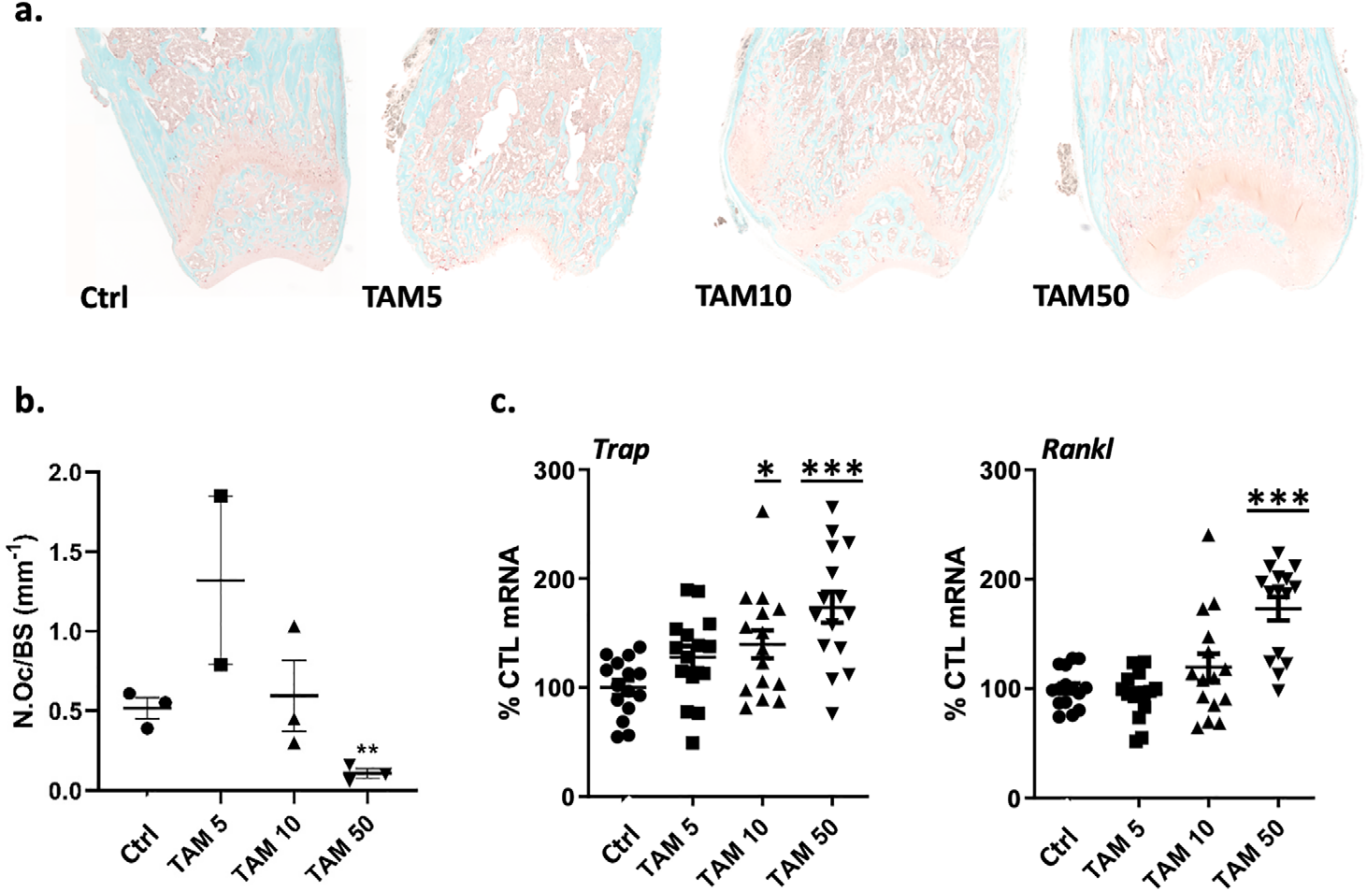
TAM effects on osteoclast number/bone surface and expression of TRAP and RANKL in C57BL/6 male mice. (*A*) Osteoclasts were stained for TRAP in tibias. (*B*) Static histomorphometric quantification demonstrates mean osteoclast numbers relative to bone surface (±SD). (*C*) Real‐time PCR for osteoclast marker genes TRAP and RANKL with RNA from tibia. Statistical significance is indicated as follows: *p < .05, **p < 0.01, and ***p < 0.001.

Last, we also examined whether the anabolic effect of TAM was potentiated by a suppression of osteoclasts, as has been shown.^(^ ^7^, ^26^ ^)^ Femurs of 4‐week‐old male C57BL/6 mice dosed with TAM were stained for TRAP: in Fig. 6, at the lowest dose (20 mg/kg total), osteoclast numbers increased by 1.6‐fold. However, at the highest dose of TAM (200 mg/kg total) there were significantly fewer osteoclasts (N.Oc/BS), less than 20% comparing to numbers in Ctrl bones (Fig. 6*A*,*B*, Supplemental Fig. 6). Analysis of mRNA for *Trap* and *Rankl* revealed that both genes were upregulated threefold at high dose (200 mg/kg total), despite the decreased osteoclast numbers on the bone surface (Fig. 6*C*). Consistent with the tight biological coupling between bone accumulation and resorption, this result suggests that osteoclasts may increase concurrently with increased osteoblast‐mediated bone formation in response to TAM and that the higher TAM dose ultimately inhibited osteoclast differentiation.

## Discussion

For investigation of bone metabolism, different promoters that drive osteogenic gene expression in bone, combined with the Cre‐loxP system, have been used to study the roles of bone‐specific genes during bone development including Col1,^(^ ^27^ ^)^ Col2,^(^ ^28^, ^29^, ^30^ ^)^ Osx,^(31^ ^)^ and Prrx1, which is specifically expressed in MSC which drive long‐bone osteoblast formation^(^ ^10^ ^)^ and which we utilized in our experiments. An inducible Cre system activated by cell‐specific regulatory elements such as the estrogen receptor (ER) further allows temporal induction through the use of exogenous inducers such as TAM, and has become a broadly applied technique.^(^ ^3^, ^4^, ^32^, ^33^, ^34^, ^35^ ^)^ Although it is well established that TAM acts as an agonist with respect to estrogen's stimulatory action on both cortical and trabecular bone accrual,^(^ ^8^, ^36^ ^)^ many laboratories have continued to utilize this system. Previous studies have utilized TAM doses between 70 and 100 mg/kg in three to five injections to achieve total doses of 225 to 500 mg/kg for validation of genetic loss‐of‐function results.^(^ ^10^, ^11^, ^12^, ^13^, ^14^, ^15^, ^16^, ^17^, ^18^, ^19^ ^)^ However, because recent work suggested a minimal effect of TAM at 10 mg/kg × 4 (40 mg/kg total),^(^ ^9^ ^)^ we began with an intermediate TAM dose (30 mg/kg, total 150 mg/kg) to induce conditional knockout of β‐catenin.

We found that bone formation was potently increased in young male mice despite excision of β‐catenin using TAM at 150 mg/kg total given in five doses. Data from μCT, histologic staining, and real‐time PCR all strongly supported this effect. Similar results were seen in male mice (Prrx1‐Cre/ERT2). As such, TAM's bone stimulatory effect at the ER overcame any potential effects due to deletions of β‐catenin. Reduction of the total dose of TAM to 60 mg/kg still induced significant increases of osteogenic parameters. Importantly, the effect of TAM was dose‐dependent, with TAM administration delivered at only a total dose of 20 mg/kg generating a significant upregulation of structural bone parameters. That we did not measure serum levels of TAM is a limitation of our work, but TAM concentration is likely to be highest 1 week after dosing^(^ ^37^ ^)^; because we dosed at least twice weekly, as is common in transgenic strategies, we likely achieved peak concentrations through the week following the final dose. Importantly, TAM potently induced bone formation in the multiple mouse strains, and in both female and male mice, used in our studies.

As a potent ER ligand, TAM is also expected to inhibit bone resorption.^(^ ^7^, ^26^, ^38^, ^39^, ^40^ ^)^ TAM directly targets apoptosis in osteoclasts^(^ ^39^ ^)^ and inhibits both Runx2‐driven transcription and Runx2‐mediated osteoblast‐driven osteoclastogenesis.^(^ ^41^ ^)^ The inhibitory effect of TAM on formation of human osteoclasts in vitro is observed at the concentrations as low as 0.01μM.^(^ ^7^ ^)^ In our in vivo study, histology showed that the highest TAM dose (200 mg/kg total) significantly reduced osteoclasts (Fig. 6*A*,*B*), whereas the lowest dose (20 mg/kg total) was associated with increased osteoclast numbers. It is likely that such osteoclast formation represents a response to increased bone turnover.^(^ ^42^ ^)^ Real‐time PCR showed a trend to increased expression of *Trap* and *Rankl* genes associated with increased formation at all TAM doses (Fig. 6*C*), but the highest dose of TAM appeared sufficient to shut down osteoclast formation. Because it is well accepted that the processes of osteoclastic bone resorption and osteoblastic bone formation are coupled to ensure an adaptive skeletal response in terms of skeletal mass,^(^ ^41^ ^)^ we were not surprised to find increased osteoclasts during TAM‐induced bone formation. Similarly, it is known that estrogen prevents osteoclastic bone resorption. Our analysis was limited by sample number, but we believe that our main point, that the highest TAM exposure significantly suppressed osteoclast numbers as shown in Fig. 6*B*, contributes to the increased bone measured. Although we thus postulate that osteoclast suppression contributes to the increased bone formation with higher doses of tamoxifen, it is not possible to assign exact values to formation and resorption in this experiment. Our results do, however, allow us to strongly caution that changes in bone metabolism in transgenic mice after temporal induction of genes will be substantially affected by TAM itself.

Previous studies show that attainment of skeletal maturity is reached between 3 and 6 months in mice strains.^(^ ^43^ ^)^ Because the mice commonly used for TAM‐induced conditional gene knockout are between 4 and 12 weeks of age, our study should be useful for considering effect of gene knockout in the mature skeleton. Interestingly, some CreERT models used mice as old as 6 months or even older, eg, Acan‐CreERT,^(^ ^44^ ^)^ when treated with tamoxifen. In cases of late exposure to TAM, we might predict that anabolic effects would be lessened as femoral cancellous and vertebral trabecular bone mass decline beginning at about 3 months of age in C57BL/6 mice.^(^ ^45^, ^46^, ^47^ ^)^ As such, it would be useful to investigate TAM effects on bone metabolism of aged mice.

TAM is widely utilized to induce a multiplicity of Cre‐drivers in genetic studies, including recent studies utilizing diets delivering TAM at 40 to 80 mg/kg/day.^(^ ^48^ ^)^ It is worth noting that our *highest* total cumulative TAM dose (200 mg/kg total) was substantially lower than doses routinely used to promote conditional transgenic strategies. Because even much lower doses of TAM strongly induce bone formation, as well as affect osteoclast numbers, we conclude that use of TAM to induce a CreER system will confound interpretations that correlate gene effects with bone metabolism. We therefore urge caution with any interpretation of bone indices or modulations in bone parameters in mice dosed with TAM. From a more constructive perspective, studies using TAM for Cre induction should properly account for TAM effects in Cre‐positive littermates with WT and/or heterozygous alleles. If results do not show a difference with the homozygous conditional KO mice, then this result will remain inconclusive because of the potent stimulatory effects of TAM. However, insights in gene function may still be obtained in this mouse model for “TAM‐dependent bone accrual,” provided that the inhibitory effects of the conditional gene knockout are more powerful than the positive effects of TAM.

## Disclosures

None of the authors have any conflicting relationships to disclose.

## Author Contributions

**Zhihui Xie:** Conceptualization; data curation; formal analysis; investigation; methodology; validation; writing‐original draft; writing‐review and editing. **Cody McGrath:** Data curation; formal analysis; investigation; methodology; validation; writing‐original draft; writing‐review and editing. **Jeyantt Sankaran:** Writing‐review and editing. **Maya Styner:** Conceptualization; funding acquisition; project administration; supervision; writing‐review and editing. **Sarah Little‐Letsinger:** Data curation; methodology; writing‐review and editing. **Amel Dudakovic:** Conceptualization; data curation; formal analysis; methodology; writing‐original draft; writing‐review and editing. **Andre van Wijnen:** Conceptualization; funding acquisition; project administration; resources; supervision; writing‐review and editing.

### Peer Review

The peer review history for this article is available at https://publons.com/publon/10.1002/jbm4.10450.

## Supporting information


**Appendix S1**. Supporting Information.
