# Supplementary material for: Low‐Dose Tamoxifen Induces Significant Bone Formation in Mice
Source: JBMR Plus. 2021 Jan 20;5(3):e10450. doi: 10.1002/jbm4.10450 (PMC7990151; doi:10.1002/jbm4.10450)

Supplemental 1 (s1). The activity of Cre recombinase of Prrx1-Cre/ERT2 was determined by crossing to Cre reporter strains, Rosa26^{LacZ} (Prrx1-Cre/Rosa26^{LacZ})

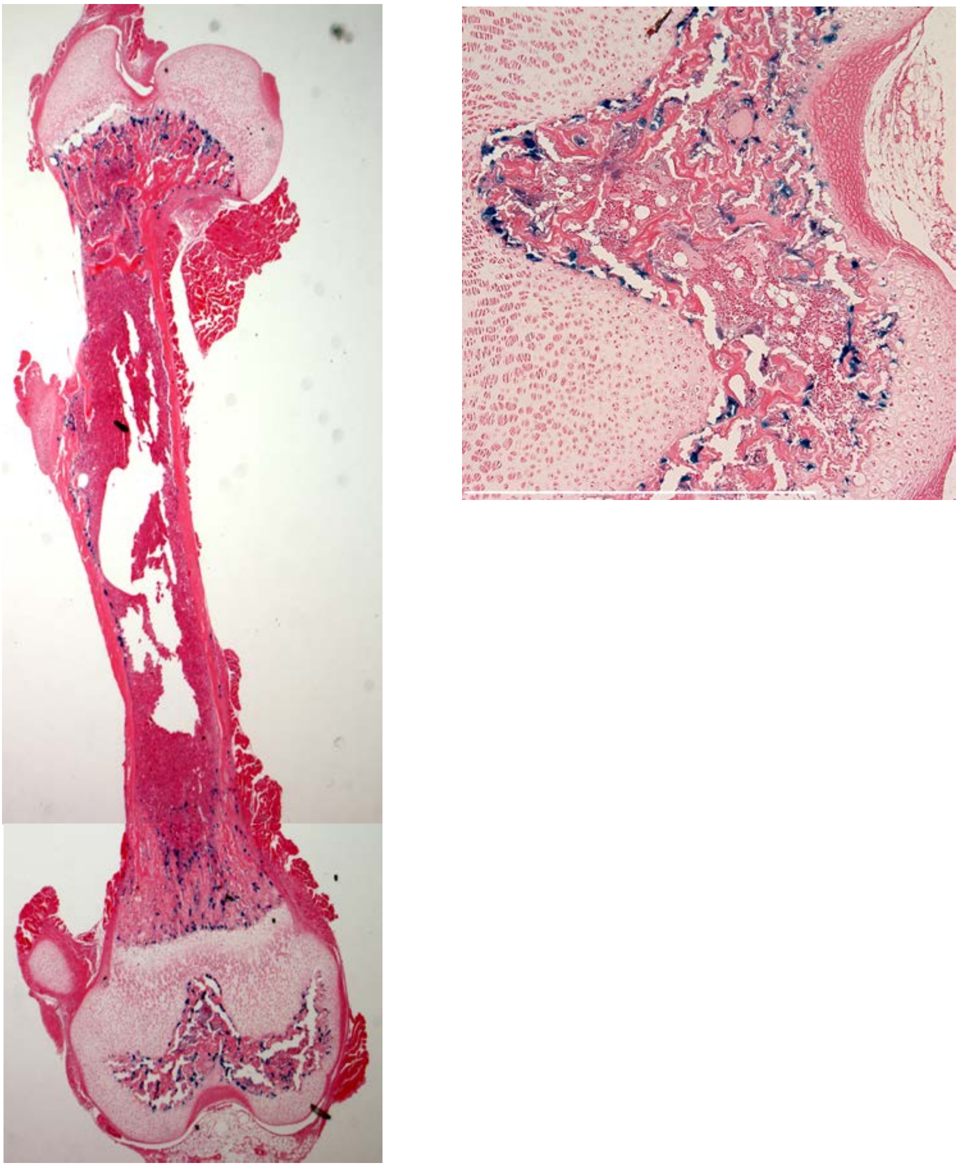

Supplemental 2 (s2). Prrx1-Cre/βCat$^{fl/fl}$ mice with 5 injections of tamoxifen

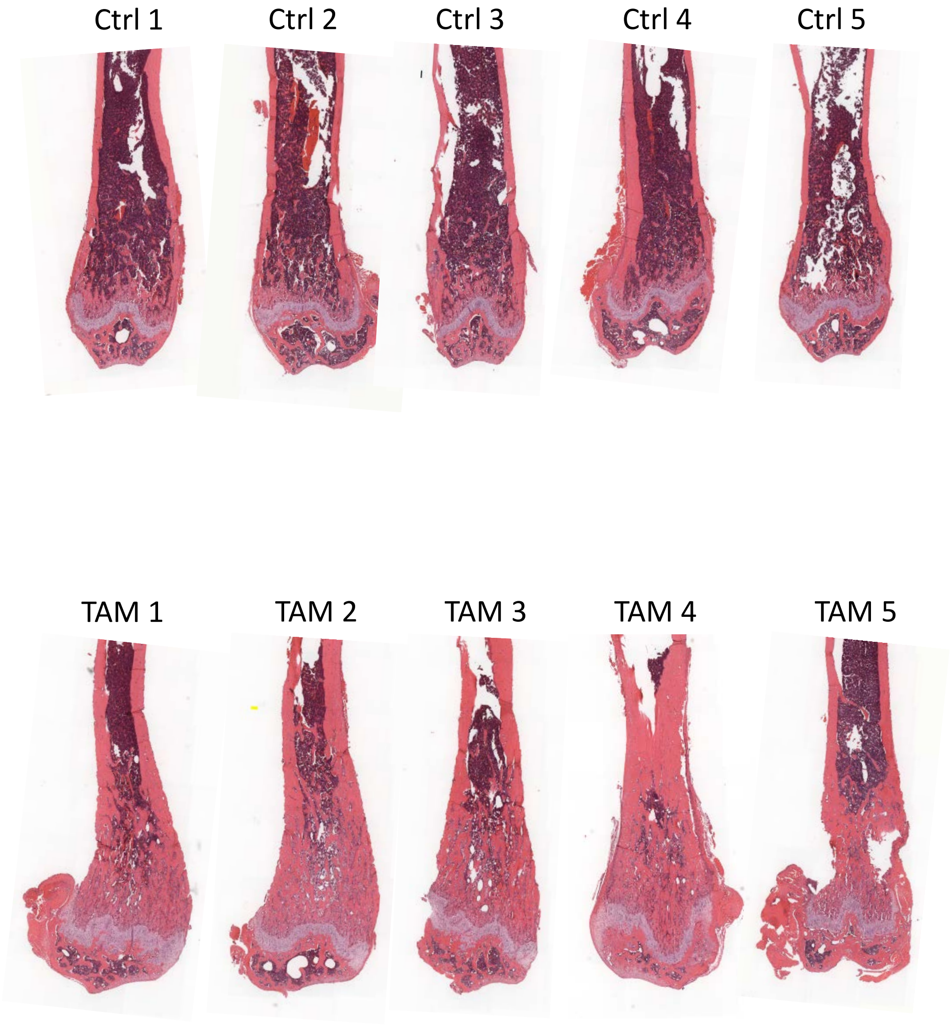

Supplemental 3 (s3). Prrx1-Cre/ERT2 mice with 5 injections of tamoxifen

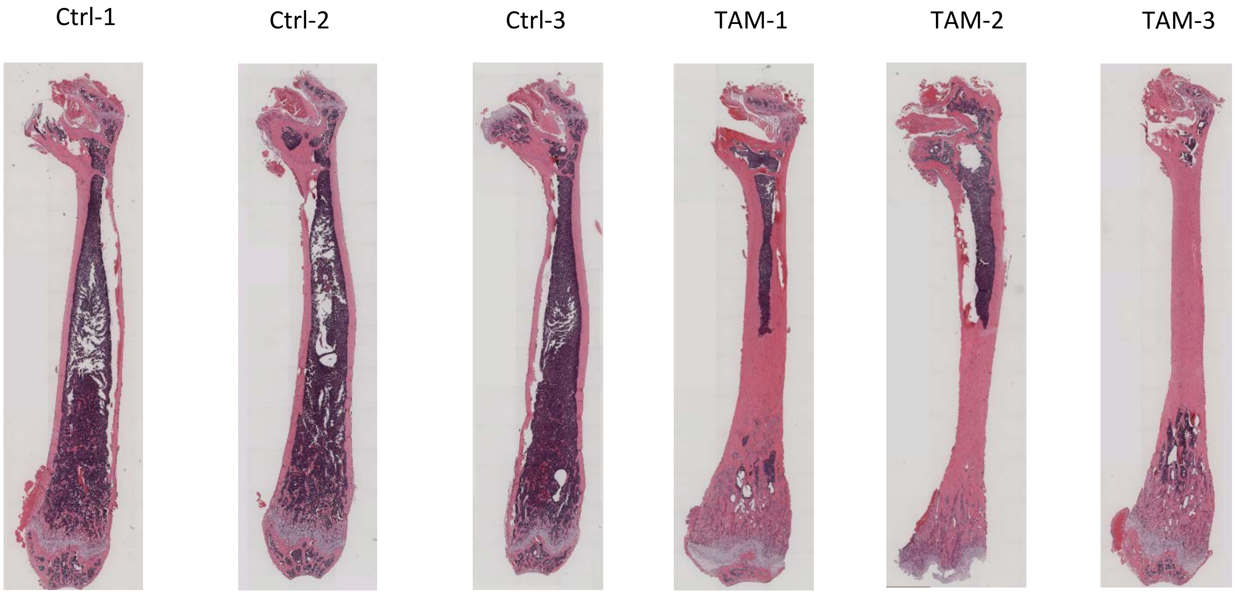

Supplemental 4 (s4). Prrx1-Cre/ERT2 mice with 2 injections of tamoxifen

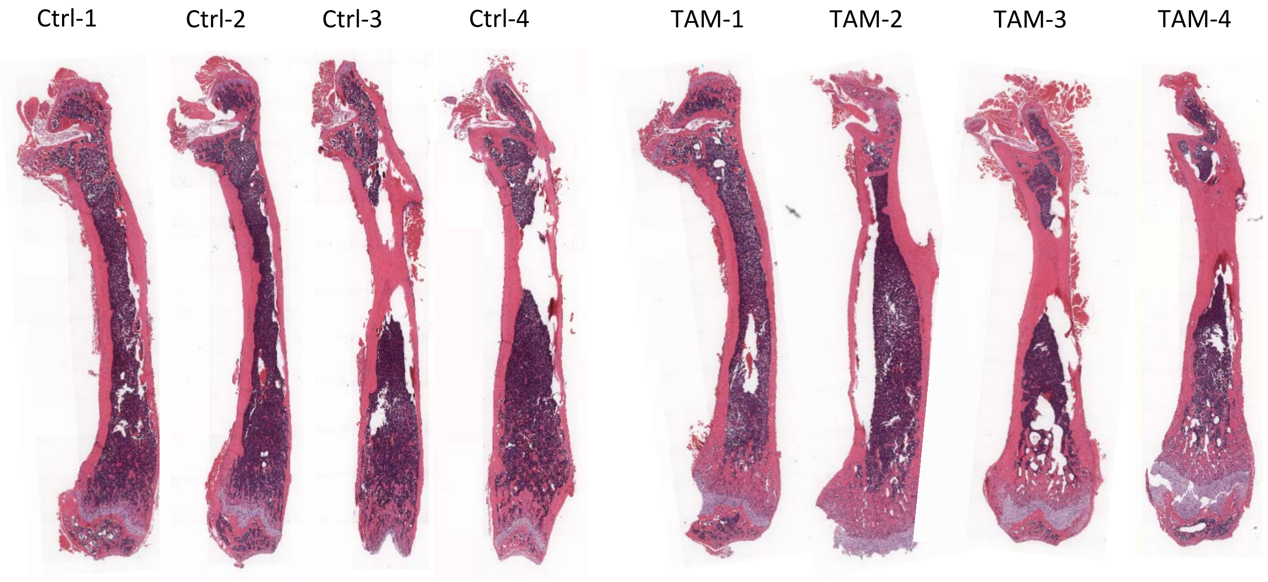

Supplemental 5 (s5). C57BL/6 mice with 3 different doses of tamoxifen (each dose x 4 injections)

H&E Staining

Ctrl

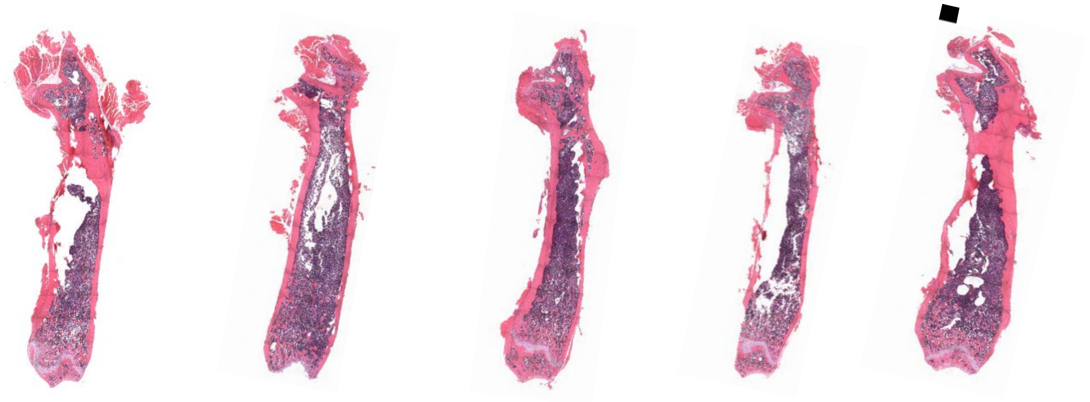

TAM (5mg/kg)

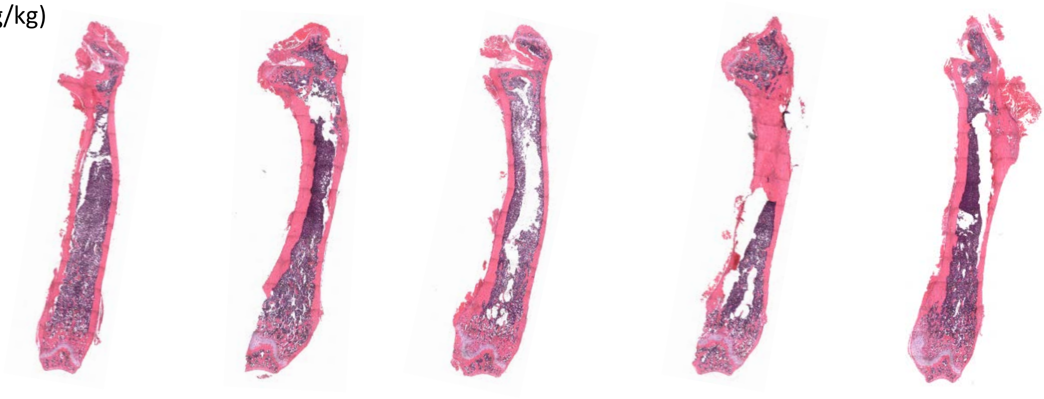

TAM (10mg/kg)

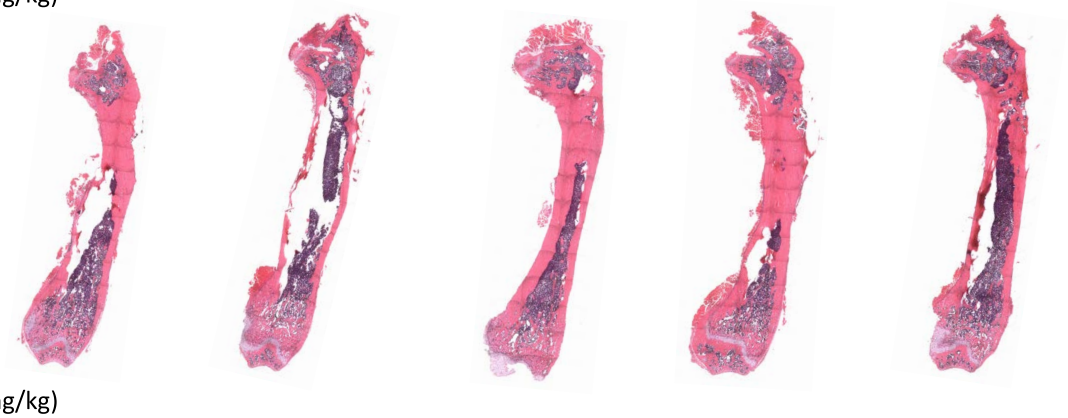

TAM (50mg/kg)

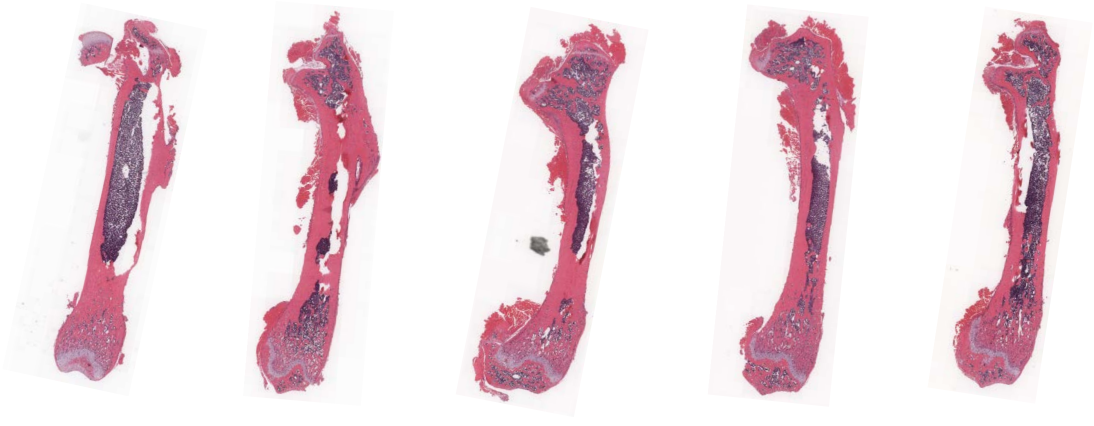

Supplemental 6 (s6). C57BL/6 mice with 3 different doses of tamoxifen (each dose x 4 injections)

TRAP Staining

Ctrl

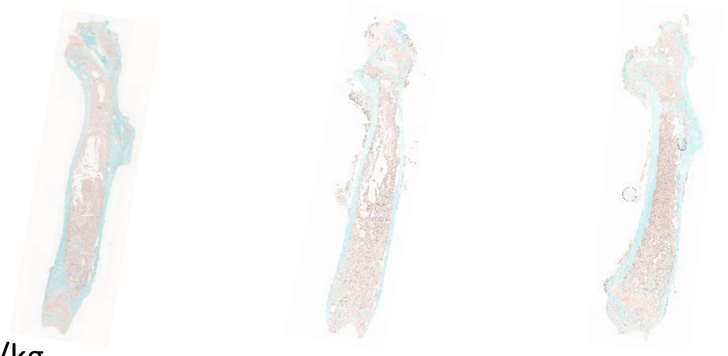

TAM 5 mg/kg

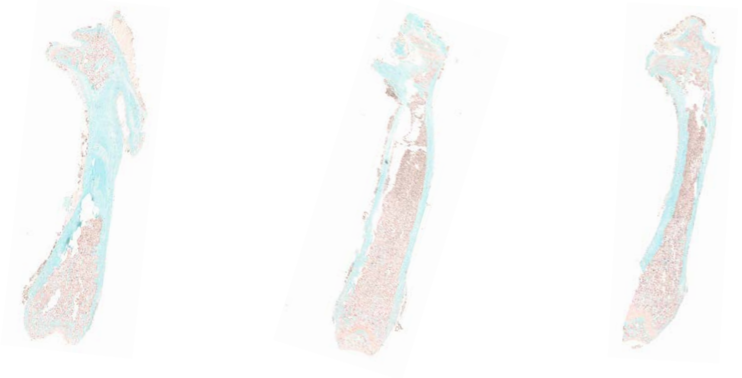

TAM 10 mg/kg

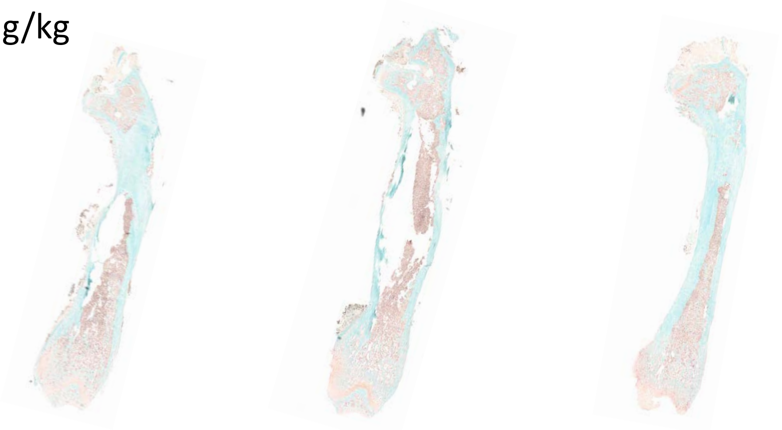

TAM 50 mg/kg

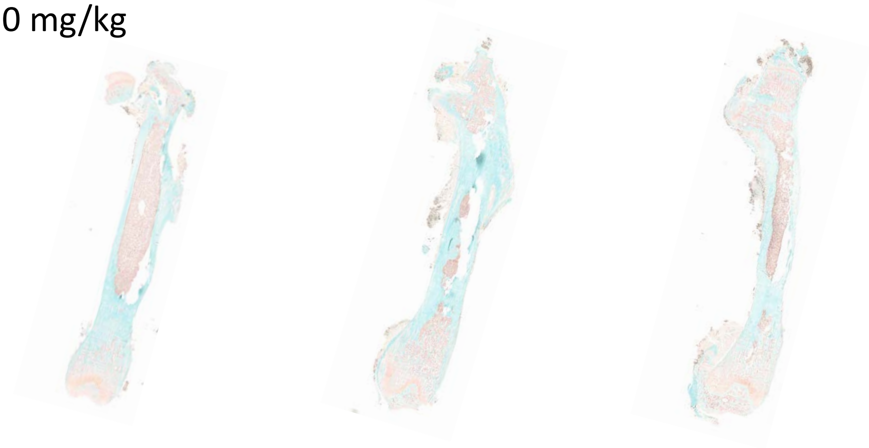

Supplement: Supplementary file 1 — Appendix S1. Supporting Information. [file JBM4-5-e10450-s001.pdf]
